# (*N*-Benzyl-*N*-isopropyl­dithio­carbamato)chloridodiphenyl­tin(IV)

**DOI:** 10.1107/S1600536810031636

**Published:** 2010-08-11

**Authors:** Amirah Faizah Abdul Muthalib, Ibrahim Baba, Mohamed Ibrahim Mohamed Tahir, Seik Weng Ng, Edward R. T. Tiekink

**Affiliations:** a School of Chemical Sciences and Food Technology, Faculty of Science and Technology, Universiti Kebangbaan Malaysia, 43600 Bangi, Malaysia; b Department of Chemistry, Universiti Putra Malaysia, 43400 Serdang, Malaysia; c Department of Chemistry, University of Malaya, 50603 Kuala Lumpur, Malaysia

## Abstract

The Sn^IV^ atom in the title organotin dithio­carbamate, [Sn(C_6_H_5_)_2_(C_11_H_14_NS_2_)Cl], is penta-coordinated by an asymmetrically coordinating dithio­carbamate ligand, a Cl and two *ispo*-C atoms of the Sn-bound phenyl groups. The resulting C_2_ClS_2_ donor set defines a coordination geometry inter­mediate between square-pyramidal and trigonal-bipyramidal with a slight tendency towards the latter. The formation of close intra­molecular C–H⋯Cl and C–H⋯S contacts precludes the Cl and S atoms from forming significant inter­molecular contacts. The presence of C–H⋯π contacts leads to the formation of supra­molecular arrays that stack along the *b* axis.

## Related literature

For a review on the applications and structural chemistry of tin dithio­carbamates, see: Tiekink (2008 ▶). For additional structural analysis, see: Addison *et al.* (1984 ▶); Spek (2009 ▶).
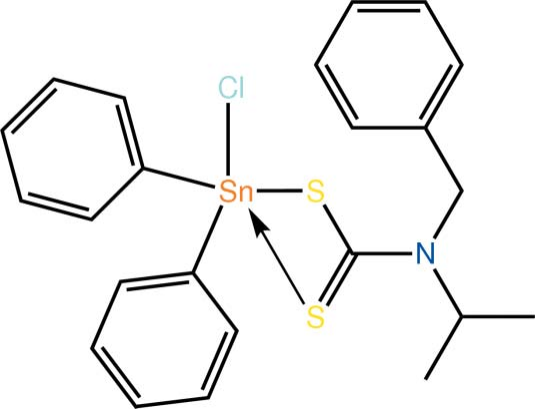


## Experimental

### 

#### Crystal data

[Sn(C_6_H_5_)_2_(C_11_H_14_NS_2_Cl]
*M* *_r_* = 532.69
Monoclinic, 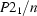

*a* = 9.1934 (1) Å
*b* = 15.1720 (2) Å
*c* = 16.8740 (2) Å
β = 96.497 (1)°
*V* = 2338.51 (5) Å^3^
*Z* = 4
Mo *K*α radiation
μ = 1.39 mm^−1^
*T* = 100 K
0.30 × 0.25 × 0.20 mm

#### Data collection

Oxford Diffraction Xcaliber Eos Gemini diffractometer
Absorption correction: multi-scan (*CrysAlis PRO*; Oxford Diffraction, 2010 ▶) *T*_min_ = 0.887, *T*_max_ = 1.000
54748 measured reflections
5345 independent reflections
4746 reflections with *I* > 2σ(*I*)
*R*_int_ = 0.042

#### Refinement

*R*[*F* ^2^ > 2σ(*F* ^2^)] = 0.017
*wR*(*F* ^2^) = 0.045
*S* = 1.04
5345 reflections
253 parameters
H-atom parameters constrained
Δρ_max_ = 0.33 e Å^−3^
Δρ_min_ = −0.33 e Å^−3^

### 

Data collection: *CrysAlis PRO* (Oxford Diffraction, 2010 ▶); cell refinement: *CrysAlis PRO*; data reduction: *CrysAlis PRO*; program(s) used to solve structure: *SHELXS97* (Sheldrick, 2008 ▶); program(s) used to refine structure: *SHELXL97* (Sheldrick, 2008 ▶); molecular graphics: *ORTEP-3* (Farrugia, 1997 ▶) and *DIAMOND* (Brandenburg, 2006 ▶); software used to prepare material for publication: *publCIF* (Westrip, 2010 ▶).

## Supplementary Material

Crystal structure: contains datablocks global, I. DOI: 10.1107/S1600536810031636/su2203sup1.cif

Structure factors: contains datablocks I. DOI: 10.1107/S1600536810031636/su2203Isup2.hkl

Additional supplementary materials:  crystallographic information; 3D view; checkCIF report

## Figures and Tables

**Table 1:** Hydrogen-bond geometry (Å, °) *Cg*1 is the centroid of the C7–C12 ring.

*D*—H⋯*A*	*D*—H	H⋯*A*	*D*⋯*A*	*D*—H⋯*A*
C2—H2⋯Cl1	0.95	2.80	3.4477 (17)	126
C6—H6⋯S2	0.95	2.80	3.4996 (16)	131
C14—H14⋯S2	1.00	2.51	3.0291 (15)	112
C3—H3⋯*Cg*1^i^	0.95	2.92	3.8002 (18)	154
C16—H16c⋯*Cg*1^ii^	0.98	2.81	3.4512 (18)	124

Symmetry codes: (i) 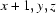; (ii) 
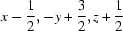
.

**Fig. 1.**
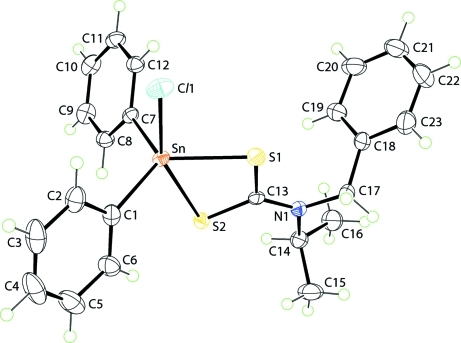
The molecular structure of the title complex showing the atom-labelling scheme and displacement ellipsoids at the 50% probability level.

**Fig. 2.**
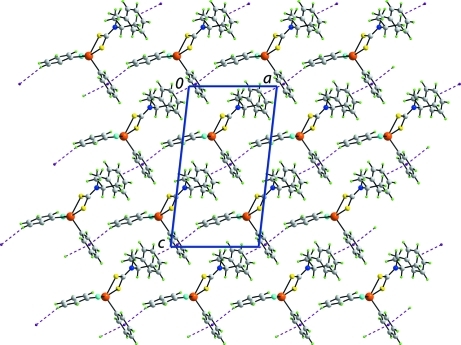
A view of the 2-D supramolecular array in the ac plane of the crystal structure of the title complex, with the C–H···π contacts shown as purple dashed lines.

**Fig. 3.**
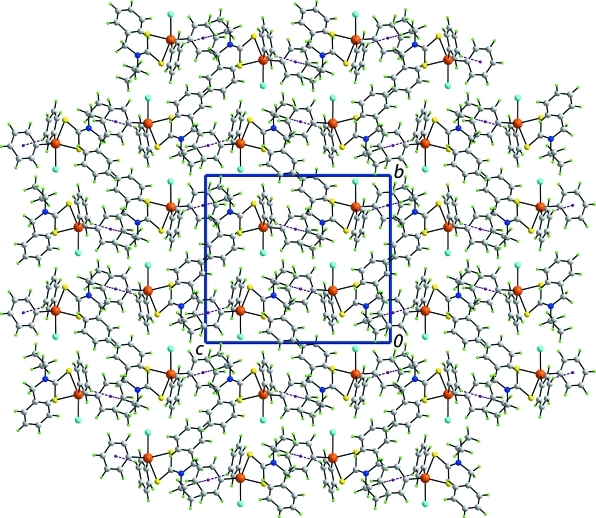
A view in projection down the a axis of the crystal structure of the title complex, showing the stacking of the 2-D arrays along the b axis. The C–H···π contacts are shown as purple dashed lines.

## supplementary crystallographic information

### Comment

Tin, including organotin, dithiocarbamates continue to attract attention as they are known to exhibit properties suggesting their potential as anti-cancer agents, anti-microbial agents and insecticides (Tiekink, 2008).

The Sn^IV^ atom in the structure of the title compound is five-coordinated, being chelated by an asymmetrically coordinating dithiocarbamate ligand, a chloride and two ispo-C atoms of the Sn-bound phenyl groups, Fig. 1. The coordination geometry is intermediate between square pyramidal and trigonal bi-pyramidal with a leaning towards the latter. This assignment is based on the value calculated for τ of 0.54 for the Sn atom, which compares to the τ values of 0.0 and 1.0 for ideal square pyramidal and trigonal bi-pyramidal geometries, respectively (Addison *et al.*, 1984; Spek, 2009). The mode of coordination of the dithiocarbamate ligand, the disposition of the ligand donor set, and the intermediate coordination geometry matches with the literature precedents (Tiekink, 2008).

The formation of close intramolecular C–H···Cl and C–H···S contacts, Table 1, preclude the Cl and S atoms from forming significant intermolecular contacts. The most prominent intermolecular interactions are of the type C–H···π, involving the Sn-bound C7–C12 ring interacting with a methyl-H on one side and a phenyl-H on the other, Fig. 2 and Table 1. This results in the formation of supramolecular arrays in the *ac* plane which stack along the *b* axis, Fig. 3.

### Experimental

Carbon disulfide (10 mmol) was added dropwise to an ethanol solution (100 ml) of *N*-benzyl-*N*-isopropyl (10 mmol). The solution was kept at 273 K for 1 h. Diphenyltin dichloride (5 mmol) dissolved in ethanol (20 ml) was added to give a white precipitate. This was collected and recrystallized from a chloroform/ethanol (1/2) mixture.

### Refinement

C-bound H-atoms were placed in calculated positions and were included in the refinement in the riding model approximation: C-H = 1.0, 0.99, 0.98 and 0.95 Å for CH, CH_2_, CH_3_ and CH(aromatic) H-atoms, respectively, with U_iso_(H) = k × U_eq_(C), where k = 1.5 for CH_3_ H-atoms, and k = 1.2 for all other H-atoms.

### Crystal data

**Table:**

[Sn(C_6_H_5_)_2_(C_11_H_14_NS_2_)Cl]	*F*(000) = 1072
*M**_r_* = 532.69	*D*_x_ = 1.513 Mg m^−^^3^
Monoclinic, *P*2_1_/*n*	Mo *K*α radiation, λ = 0.71073 Å
Hall symbol: -P 2yn	Cell parameters from 34829 reflections
*a* = 9.1934 (1) Å	θ = 2.2–29.2°
*b* = 15.1720 (2) Å	µ = 1.39 mm^−^^1^
*c* = 16.8740 (2) Å	*T* = 100 K
β = 96.497 (1)°	Block, colourless
*V* = 2338.51 (5) Å^3^	0.30 × 0.25 × 0.20 mm
*Z* = 4	

### Data collection

**Table:**

Oxford Diffraction Xcaliber Eos Gemini diffractometer	5345 independent reflections
Radiation source: fine-focus sealed tube	4746 reflections with *I* > 2σ(*I*)
graphite	*R*_int_ = 0.042
Detector resolution: 16.1952 pixels mm^-1^	θ_max_ = 27.5°, θ_min_ = 2.4°
ω scans	*h* = −11→11
Absorption correction: multi-scan (*CrysAlis PRO*; Oxford Diffraction, 2010)	*k* = −19→19
*T*_min_ = 0.887, *T*_max_ = 1.000	*l* = −21→21
54748 measured reflections	

### Refinement

**Table:**

Refinement on *F*^2^	Primary atom site location: structure-invariant direct methods
Least-squares matrix: full	Secondary atom site location: difference Fourier map
*R*[*F*^2^ > 2σ(*F*^2^)] = 0.017	Hydrogen site location: inferred from neighbouring sites
*wR*(*F*^2^) = 0.045	H-atom parameters constrained
*S* = 1.04	*w* = 1/[σ^2^(*F*_o_^2^) + (0.0285*P*)^2^] where *P* = (*F*_o_^2^ + 2*F*_c_^2^)/3
5345 reflections	(Δ/σ)_max_ = 0.001
253 parameters	Δρ_max_ = 0.33 e Å^−^^3^
0 restraints	Δρ_min_ = −0.33 e Å^−^^3^

### Fractional atomic coordinates and isotropic or equivalent isotropic displacement parameters (Å^2^)

**Table:**

	*x*	*y*	*z*	*U*_iso_*/*U*_eq_	
Sn	0.677004 (10)	0.690203 (6)	0.688075 (5)	0.01845 (4)	
Cl1	0.76007 (4)	0.53663 (2)	0.68888 (2)	0.03259 (9)	
S1	0.58215 (4)	0.65598 (2)	0.81529 (2)	0.02475 (8)	
S2	0.54077 (4)	0.83082 (2)	0.73935 (2)	0.02118 (8)	
N1	0.41603 (13)	0.78207 (8)	0.86783 (7)	0.0232 (3)	
C1	0.88272 (15)	0.75295 (10)	0.67971 (8)	0.0240 (3)	
C2	0.99547 (18)	0.70469 (11)	0.65168 (10)	0.0325 (4)	
H2	0.9832	0.6434	0.6416	0.039*	
C3	1.12574 (18)	0.74585 (15)	0.63840 (10)	0.0439 (5)	
H3	1.2024	0.7127	0.6195	0.053*	
C4	1.14323 (19)	0.83449 (15)	0.65269 (12)	0.0482 (5)	
H4	1.2318	0.8627	0.6429	0.058*	
C5	1.03361 (19)	0.88287 (13)	0.68104 (11)	0.0438 (5)	
H5	1.0469	0.9441	0.6910	0.053*	
C6	0.90314 (17)	0.84220 (11)	0.69513 (10)	0.0318 (4)	
H6	0.8281	0.8756	0.7153	0.038*	
C7	0.52428 (15)	0.68234 (8)	0.58354 (8)	0.0185 (3)	
C8	0.49266 (16)	0.75798 (10)	0.53744 (9)	0.0242 (3)	
H8	0.5373	0.8125	0.5539	0.029*	
C9	0.39670 (17)	0.75412 (11)	0.46787 (9)	0.0292 (3)	
H9	0.3767	0.8057	0.4366	0.035*	
C10	0.33020 (17)	0.67518 (11)	0.44404 (10)	0.0297 (4)	
H10	0.2646	0.6725	0.3964	0.036*	
C11	0.35952 (16)	0.59975 (10)	0.48987 (9)	0.0274 (3)	
H11	0.3130	0.5457	0.4739	0.033*	
C12	0.45682 (15)	0.60357 (9)	0.55895 (9)	0.0227 (3)	
H12	0.4775	0.5517	0.5897	0.027*	
C13	0.50196 (15)	0.76049 (9)	0.81307 (8)	0.0199 (3)	
C14	0.34197 (16)	0.86968 (10)	0.86646 (9)	0.0289 (3)	
H14	0.3399	0.8938	0.8112	0.035*	
C15	0.4319 (2)	0.93275 (12)	0.92244 (12)	0.0431 (4)	
H15A	0.5321	0.9355	0.9081	0.065*	
H15B	0.4335	0.9118	0.9775	0.065*	
H15C	0.3879	0.9916	0.9178	0.065*	
C16	0.18495 (18)	0.86220 (12)	0.88488 (11)	0.0400 (4)	
H16A	0.1320	0.8206	0.8477	0.060*	
H16B	0.1379	0.9202	0.8792	0.060*	
H16C	0.1835	0.8410	0.9397	0.060*	
C17	0.40459 (18)	0.72348 (11)	0.93638 (9)	0.0294 (3)	
H17A	0.3741	0.7592	0.9808	0.035*	
H17B	0.5030	0.6994	0.9541	0.035*	
C18	0.29886 (16)	0.64710 (10)	0.92112 (9)	0.0249 (3)	
C19	0.20502 (18)	0.63709 (11)	0.85206 (9)	0.0316 (3)	
H19A	0.2080	0.6781	0.8097	0.038*	
C20	0.1059 (2)	0.56752 (11)	0.84380 (12)	0.0411 (4)	
H20	0.0412	0.5614	0.7961	0.049*	
C21	0.1015 (2)	0.50811 (12)	0.90402 (13)	0.0519 (5)	
H21	0.0325	0.4612	0.8988	0.062*	
C22	0.1979 (3)	0.51627 (13)	0.97299 (13)	0.0581 (6)	
H22	0.1962	0.4743	1.0146	0.070*	
C23	0.2960 (2)	0.58509 (11)	0.98129 (10)	0.0406 (4)	
H23	0.3622	0.5901	1.0286	0.049*	

### Atomic displacement parameters (Å^2^)

**Table:**

	*U*^11^	*U*^22^	*U*^33^	*U*^12^	*U*^13^	*U*^23^
Sn	0.01680 (6)	0.01905 (6)	0.01915 (6)	0.00046 (4)	0.00050 (4)	−0.00322 (3)
Cl1	0.0362 (2)	0.02243 (19)	0.0372 (2)	0.00957 (16)	−0.00441 (17)	−0.00494 (15)
S1	0.0298 (2)	0.02159 (18)	0.02287 (18)	−0.00067 (15)	0.00296 (15)	0.00143 (14)
S2	0.02277 (18)	0.01888 (17)	0.02314 (18)	−0.00102 (13)	0.00801 (14)	−0.00170 (13)
N1	0.0239 (6)	0.0260 (6)	0.0202 (6)	−0.0069 (5)	0.0053 (5)	−0.0045 (5)
C1	0.0161 (7)	0.0360 (9)	0.0193 (7)	−0.0015 (6)	−0.0004 (5)	0.0015 (6)
C2	0.0256 (8)	0.0448 (10)	0.0275 (8)	0.0053 (7)	0.0045 (7)	0.0010 (7)
C3	0.0212 (8)	0.0748 (14)	0.0370 (10)	0.0076 (9)	0.0090 (7)	0.0106 (9)
C4	0.0207 (9)	0.0789 (15)	0.0442 (11)	−0.0136 (9)	0.0004 (8)	0.0186 (10)
C5	0.0319 (9)	0.0488 (11)	0.0491 (11)	−0.0161 (8)	−0.0028 (8)	0.0075 (9)
C6	0.0242 (8)	0.0358 (9)	0.0348 (9)	−0.0062 (7)	0.0010 (7)	−0.0008 (7)
C7	0.0158 (6)	0.0212 (7)	0.0186 (7)	0.0015 (5)	0.0014 (5)	−0.0037 (5)
C8	0.0244 (8)	0.0208 (8)	0.0274 (8)	0.0002 (6)	0.0037 (6)	−0.0017 (6)
C9	0.0291 (8)	0.0311 (9)	0.0267 (8)	0.0091 (7)	0.0007 (6)	0.0044 (6)
C10	0.0219 (8)	0.0421 (10)	0.0236 (8)	0.0054 (7)	−0.0042 (6)	−0.0049 (7)
C11	0.0212 (7)	0.0296 (8)	0.0309 (8)	−0.0034 (6)	0.0011 (6)	−0.0092 (6)
C12	0.0219 (7)	0.0206 (7)	0.0256 (7)	0.0004 (6)	0.0031 (6)	−0.0017 (6)
C13	0.0183 (7)	0.0222 (7)	0.0188 (7)	−0.0066 (6)	0.0003 (5)	−0.0053 (5)
C14	0.0297 (8)	0.0274 (8)	0.0317 (8)	−0.0026 (7)	0.0132 (7)	−0.0076 (6)
C15	0.0418 (10)	0.0331 (10)	0.0560 (12)	−0.0112 (8)	0.0128 (9)	−0.0188 (8)
C16	0.0332 (9)	0.0421 (10)	0.0477 (11)	−0.0030 (8)	0.0179 (8)	−0.0098 (8)
C17	0.0318 (8)	0.0393 (9)	0.0177 (7)	−0.0093 (7)	0.0046 (6)	−0.0023 (6)
C18	0.0271 (8)	0.0251 (8)	0.0241 (7)	−0.0007 (6)	0.0095 (6)	−0.0019 (6)
C19	0.0343 (9)	0.0287 (9)	0.0316 (8)	−0.0063 (7)	0.0026 (7)	−0.0001 (7)
C20	0.0382 (10)	0.0344 (10)	0.0516 (11)	−0.0104 (8)	0.0083 (8)	−0.0124 (8)
C21	0.0649 (13)	0.0325 (10)	0.0636 (13)	−0.0202 (9)	0.0301 (11)	−0.0113 (9)
C22	0.0979 (18)	0.0314 (10)	0.0502 (12)	−0.0109 (11)	0.0309 (12)	0.0071 (9)
C23	0.0587 (12)	0.0353 (10)	0.0288 (9)	0.0018 (9)	0.0100 (8)	0.0027 (7)

### Geometric parameters (Å, °)

**Table:**

Sn—S1	2.4621 (4)	C10—H10	0.9500
Sn—S2	2.6672 (4)	C11—C12	1.388 (2)
Sn—C1	2.1362 (14)	C11—H11	0.9500
Sn—C7	2.1300 (14)	C12—H12	0.9500
Sn—Cl1	2.4515 (4)	C14—C16	1.515 (2)
S1—C13	1.7472 (15)	C14—C15	1.521 (2)
S2—C13	1.7070 (14)	C14—H14	1.0000
N1—C13	1.3230 (18)	C15—H15A	0.9800
N1—C17	1.4721 (19)	C15—H15B	0.9800
N1—C14	1.492 (2)	C15—H15C	0.9800
C1—C6	1.388 (2)	C16—H16A	0.9800
C1—C2	1.395 (2)	C16—H16B	0.9800
C2—C3	1.391 (2)	C16—H16C	0.9800
C2—H2	0.9500	C17—C18	1.516 (2)
C3—C4	1.373 (3)	C17—H17A	0.9900
C3—H3	0.9500	C17—H17B	0.9900
C4—C5	1.376 (3)	C18—C19	1.378 (2)
C4—H4	0.9500	C18—C23	1.387 (2)
C5—C6	1.393 (2)	C19—C20	1.391 (2)
C5—H5	0.9500	C19—H19A	0.9500
C6—H6	0.9500	C20—C21	1.363 (3)
C7—C12	1.3881 (19)	C20—H20	0.9500
C7—C8	1.3984 (19)	C21—C22	1.386 (3)
C8—C9	1.388 (2)	C21—H21	0.9500
C8—H8	0.9500	C22—C23	1.377 (3)
C9—C10	1.384 (2)	C22—H22	0.9500
C9—H9	0.9500	C23—H23	0.9500
C10—C11	1.390 (2)		
			
C7—Sn—C1	118.41 (5)	C11—C12—C7	120.71 (13)
C7—Sn—Cl1	97.30 (4)	C11—C12—H12	119.6
C1—Sn—Cl1	98.42 (4)	C7—C12—H12	119.6
C7—Sn—S1	116.42 (4)	N1—C13—S2	123.01 (11)
C1—Sn—S1	123.72 (4)	N1—C13—S1	119.57 (11)
Cl1—Sn—S1	86.307 (14)	S2—C13—S1	117.42 (8)
C7—Sn—S2	91.36 (4)	N1—C14—C16	111.95 (13)
C1—Sn—S2	96.70 (4)	N1—C14—C15	109.62 (13)
Cl1—Sn—S2	156.326 (13)	C16—C14—C15	112.52 (13)
S1—Sn—S2	70.135 (12)	N1—C14—H14	107.5
C13—S1—Sn	88.86 (5)	C16—C14—H14	107.5
C13—S2—Sn	83.16 (5)	C15—C14—H14	107.5
C13—N1—C17	120.05 (13)	C14—C15—H15A	109.5
C13—N1—C14	121.13 (12)	C14—C15—H15B	109.5
C17—N1—C14	118.49 (12)	H15A—C15—H15B	109.5
C6—C1—C2	119.07 (14)	C14—C15—H15C	109.5
C6—C1—Sn	121.63 (11)	H15A—C15—H15C	109.5
C2—C1—Sn	119.07 (12)	H15B—C15—H15C	109.5
C1—C2—C3	120.35 (17)	C14—C16—H16A	109.5
C1—C2—H2	119.8	C14—C16—H16B	109.5
C3—C2—H2	119.8	H16A—C16—H16B	109.5
C4—C3—C2	119.83 (17)	C14—C16—H16C	109.5
C4—C3—H3	120.1	H16A—C16—H16C	109.5
C2—C3—H3	120.1	H16B—C16—H16C	109.5
C3—C4—C5	120.53 (17)	N1—C17—C18	115.44 (12)
C3—C4—H4	119.7	N1—C17—H17A	108.4
C5—C4—H4	119.7	C18—C17—H17A	108.4
C4—C5—C6	120.12 (18)	N1—C17—H17B	108.4
C4—C5—H5	119.9	C18—C17—H17B	108.4
C6—C5—H5	119.9	H17A—C17—H17B	107.5
C1—C6—C5	120.07 (16)	C19—C18—C23	118.87 (15)
C1—C6—H6	120.0	C19—C18—C17	123.79 (14)
C5—C6—H6	120.0	C23—C18—C17	117.33 (14)
C12—C7—C8	118.86 (13)	C18—C19—C20	120.58 (16)
C12—C7—Sn	121.74 (10)	C18—C19—H19A	119.7
C8—C7—Sn	119.39 (10)	C20—C19—H19A	119.7
C9—C8—C7	120.54 (14)	C21—C20—C19	120.13 (18)
C9—C8—H8	119.7	C21—C20—H20	119.9
C7—C8—H8	119.7	C19—C20—H20	119.9
C8—C9—C10	120.02 (15)	C20—C21—C22	119.78 (18)
C8—C9—H9	120.0	C20—C21—H21	120.1
C10—C9—H9	120.0	C22—C21—H21	120.1
C9—C10—C11	119.95 (14)	C23—C22—C21	120.17 (18)
C9—C10—H10	120.0	C23—C22—H22	119.9
C11—C10—H10	120.0	C21—C22—H22	119.9
C12—C11—C10	119.92 (14)	C22—C23—C18	120.43 (18)
C12—C11—H11	120.0	C22—C23—H23	119.8
C10—C11—H11	120.0	C18—C23—H23	119.8
			
C7—Sn—S1—C13	−77.41 (6)	C12—C7—C8—C9	−0.7 (2)
C1—Sn—S1—C13	88.60 (7)	Sn—C7—C8—C9	178.20 (11)
Cl1—Sn—S1—C13	−173.74 (5)	C7—C8—C9—C10	0.7 (2)
S2—Sn—S1—C13	3.88 (4)	C8—C9—C10—C11	0.0 (2)
C7—Sn—S2—C13	113.69 (6)	C9—C10—C11—C12	−0.8 (2)
C1—Sn—S2—C13	−127.49 (6)	C10—C11—C12—C7	0.8 (2)
Cl1—Sn—S2—C13	1.93 (6)	C8—C7—C12—C11	−0.1 (2)
S1—Sn—S2—C13	−4.00 (5)	Sn—C7—C12—C11	−178.90 (11)
C7—Sn—C1—C6	91.75 (12)	C17—N1—C13—S2	−170.93 (10)
Cl1—Sn—C1—C6	−165.18 (11)	C14—N1—C13—S2	2.41 (18)
S1—Sn—C1—C6	−74.01 (12)	C17—N1—C13—S1	8.53 (17)
S2—Sn—C1—C6	−3.45 (12)	C14—N1—C13—S1	−178.13 (10)
C7—Sn—C1—C2	−82.83 (13)	Sn—S2—C13—N1	−174.56 (12)
Cl1—Sn—C1—C2	20.24 (12)	Sn—S2—C13—S1	5.98 (7)
S1—Sn—C1—C2	111.41 (11)	Sn—S1—C13—N1	174.08 (11)
S2—Sn—C1—C2	−178.04 (11)	Sn—S1—C13—S2	−6.43 (7)
C6—C1—C2—C3	−0.8 (2)	C13—N1—C14—C16	137.97 (14)
Sn—C1—C2—C3	173.88 (12)	C17—N1—C14—C16	−48.59 (18)
C1—C2—C3—C4	−0.2 (3)	C13—N1—C14—C15	−96.44 (16)
C2—C3—C4—C5	0.8 (3)	C17—N1—C14—C15	77.01 (16)
C3—C4—C5—C6	−0.3 (3)	C13—N1—C17—C18	−82.58 (17)
C2—C1—C6—C5	1.3 (2)	C14—N1—C17—C18	103.90 (16)
Sn—C1—C6—C5	−173.25 (12)	N1—C17—C18—C19	−7.8 (2)
C4—C5—C6—C1	−0.8 (3)	N1—C17—C18—C23	173.37 (14)
C1—Sn—C7—C12	130.17 (12)	C23—C18—C19—C20	2.0 (2)
Cl1—Sn—C7—C12	26.46 (12)	C17—C18—C19—C20	−176.85 (15)
S1—Sn—C7—C12	−63.03 (12)	C18—C19—C20—C21	−0.4 (3)
S2—Sn—C7—C12	−131.46 (11)	C19—C20—C21—C22	−1.2 (3)
C1—Sn—C7—C8	−48.66 (13)	C20—C21—C22—C23	1.2 (3)
Cl1—Sn—C7—C8	−152.38 (11)	C21—C22—C23—C18	0.4 (3)
S1—Sn—C7—C8	118.13 (11)	C19—C18—C23—C22	−2.0 (3)
S2—Sn—C7—C8	49.71 (11)	C17—C18—C23—C22	176.94 (17)

### Hydrogen-bond geometry (Å, °)

**Table:**

Cg1 is the centroid of the C7–C12 ring.

**Table:**

*D*—H···*A*	*D*—H	H···*A*	*D*···*A*	*D*—H···*A*
C2—H2···Cl1	0.95	2.80	3.4477 (17)	126
C6—H6···S2	0.95	2.80	3.4996 (16)	131
C14—H14···S2	1.00	2.51	3.0291 (15)	112
C3—H3···Cg1^i^	0.95	2.92	3.8002 (18)	154
C16—H16c···Cg1^ii^	0.98	2.81	3.4512 (18)	124

Symmetry codes:  (i) *x*+1, *y*, *z*; (ii) *x*−1/2, −*y*+3/2, *z*+1/2.
